# Amphitremida (Poche, 1913) Is a New Major, Ubiquitous Labyrinthulomycete Clade

**DOI:** 10.1371/journal.pone.0053046

**Published:** 2013-01-14

**Authors:** Fatma Gomaa, Edward A. D. Mitchell, Enrique Lara

**Affiliations:** 1 Laboratory of Soil Biology, University of Neuchâtel, Neuchâtel, Switzerland; 2 Zoology Department, Faculty of Science, Ain Shams University, Cairo, Egypt; George Washington University, United States of America

## Abstract

Micro-eukaryotic diversity is poorly documented at all taxonomic levels and the phylogenetic affiliation of many taxa – including many well-known and common organisms - remains unknown. Among these *incertae sedis* taxa are *Archerella flavum* (Loeblich and Tappan, 1961) and *Amphitrema wrightianum* (Archer, 1869) (Amphitremidae), two filose testate amoebae commonly found in *Sphagnum* peatlands. To clarify their phylogenetic position, we amplified and sequenced the SSU rRNA gene obtained from four independent DNA extractions of *A. flavum* and three independent DNA extractions of *A. wrightianum.* Our molecular data demonstrate that genera *Archerella* and *Amphitrema* form a fully supported deep-branching clade within the Labyrinthulomycetes (Stramenopiles), together with *Diplophrys* sp. (ATCC50360) and several environmental clones obtained from a wide range of environments. This newly described clade we named Amphitremida is diverse genetically, ecologically and physiologically. Our phylogenetic analysis suggests that osmotrophic species evolved most likely from phagotrophic ancestors and that the bothrosome, an organelle that produces cytoplasmic networks used for attachment to the substratum and to absorb nutrients from the environments, appeared lately in labyrithulomycete evolution.

## Introduction

Molecular phylogenetic studies have revealed a tremendous diversity within unicellular eukaryotes, and the existence of ca. 55 major eukaryotic lineages [1], [2]. Furthermore, recent environmental DNA studies are continuously revealing novel clades, often comprising pico-sized <2–3 µm microorganisms lacking conspicuous morphological features [3], [4]. However, the proper assessment of eukaryotic diversity and the accurate reconstruction of the eukaryote phylogeny are hindered by the unresolved phylogenetic position of many taxa, including abundant and morphologically easily identifiable ones [5], [6]. These organisms, referred to as “*incertae sedis*” include several amoeboid eukaryotic groups, among which unusual testate amoebae belonging to family Amphitremidae [7].

**Figure 1:**
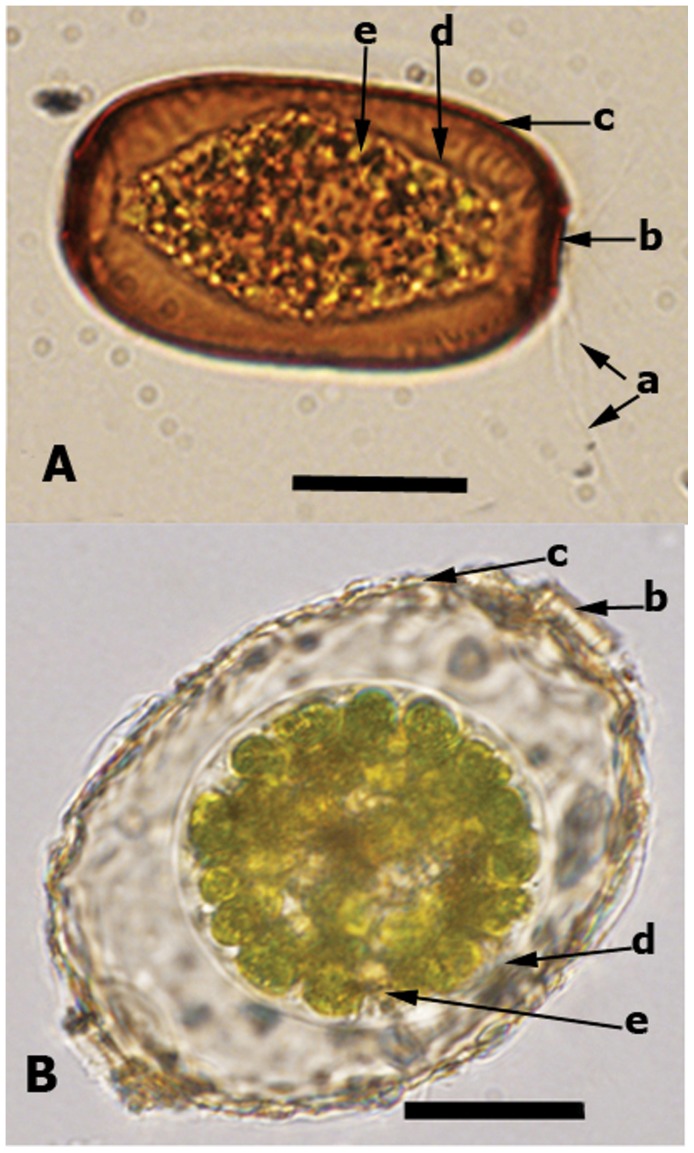
Light microscopy photograph for *Archerella flavum* (A) and *Amphitrema wrightianum* (B): the arrows indicate a) filose pseudopodia; b) pseudostome (shell aperture); c) shell (test); d) cell membrane; e) endosymbioitic green algae. Scale bar = 20 µm.

Amphitremidae are single-celled eukaryotes characterized by the presence of a shell (test) with two apertures (pseudostomes) at the opposite ends of the shell. It includes the genera *Amphitrema, Archerella* and *Paramphitrema* [8] (Table 1). The first two genera include organisms that possess filamentous and sometimes anastomosing pseudopodia, and harbor endosymbiotic zoochlorellae (Figure 1), while *Paramphitrema* lives on marine and freshwater plants and algae, and is has linear pseudopodia; its classification within Amphitremidae is debatable [8]. *Amphitrema* and *Archerella* are found primarily in *Sphagnum* peatlands and are considered as excellent bioindicators of surface moisture and water chemistry [9], [10]. These taxa are also frequently recovered as microfossils from peat deposits and are therefore useful for palaeoenvironmental reconstructions [11], [12].

**Table 1:** General characteristics of the four genera of Amphitremida.

	*Amphitrema wrightianum* (Archer, 1869)	*Archerella flavum* (Loeblich and Tappan, 1961)	*Diplophrys* sp. (Barker, 1868)	*Paramphitrema* sp. (Lauterborn, 1895)
**Shell (test) shape**	Test elliptical or lemon like shape with convex sides and two pseudostome at the opposite sides	Test elliptical, rigid, and compressed with parallel sides and two pseudostome at the opposite sides	Spherical shape and thin with two pseudostomes at the opposite sides	Test elliptical, compressed, convex sides and two pseudostome at the opposite sides
**Shell structure**	Inner organic layer and outer agglutinated (xenosomes) layer	Organic 3 layers, no xenosomes	Organic	Agglutinated (xenosomes)
**Types of filopodia**	Several thin branched filopodia	Several thin branched filopodia	Numerous long radiating, very thin branched filopodia	Two different pseudopodia (at one side one long thick unbranched and at the opposite side thin and branched
***Zoochlorella***	Present	Present	Absent	Absent
**Habitat**	Wet to submerged *Sphagnum* mosses, in peat bog pools	Moist to wet *Sphagnum* mosses, in peat bog hollows and wet lawns	Submerged marine and freshwater plants and algae	Submerged marine and freshwater plants and algae

Taxonomical placement of genera *Amphitrema* and *Archerella* has always been problematic. Penard [13] included genus *Archerella* within *Amphitrema* and described *Amphitrema flavum* as a “Thecamoebidae” with a filamentous pseudopodia and rich with *zoochlorellae* endosymbiont. Later, Wailes [14] created a new clade for filamentous amoebae with two apertures on the test, that he called Amphistomina, and that comprised genera *Amphitrema* and *Diplophrys* [15], but doubted on the validity of this taxon, where members shared only the double aperture as common feature. This view was however supported by De Sandeleer [16] who placed all these organisms within Granuloreticulosea (roughly equal to Foraminifera sensu Adl et al. [17]) based on their branched and anastomosing pseudopodia (Table 1). However, later analyses revealed that true foraminiferans are characterized by the presence of granular pseudopods also called granuloreticulopodia [18] that exhibit a typical bidirectional protoplasmic streaming [19]. Bonnet et al., [20] described the ultrastructure of *Amphitrema* ( =  *Archerella*) *flavum* and its tubulocristate mitochondria; such structures are repeatedly found within “core Cercozoa” [2], suggesting a relationship with filose amoebae such as for instance the Euglyphida. However, similar structures were also found in stramenopiles [17] or in totally unrelated organisms such as jakobids [21]. In the recent literature, *Amphitrema* and *Archerella* are considered as forming part of a single family, the Amphitremidae, together perhaps with the enigmatic *Paramphitrema.* They have been placed as testate amoebae with filopodia *incertae sedis* [22]. Their position remained unsolved by the time of the publication of Adl et al’s revision of all micro-eukaryotic taxonomy [17], genus *Amphitrema* remained amongst the protist genera with uncertain affiliation.

In order clarify their phylogenetic position within the tree of eukaryote; we performed the first molecular study based on SSU rRNA gene sequences in the two most common genera of Amphitremidae, *Archerella* and *Amphitrema.* In a second step, we performed a search in GenBank to assess the environmental diversity of this clade and the variety of environments colonized.

## Materials and Methods

### Samples Collection and Documentation

We sampled *Archerella flavum* and *Amphitrema wrightianum* (Figure 1) from wet *Sphagnum* mosses collected from the west shore of Duffey Lake, South Central British Columbia, Canada (50°23′ N 122°27′ W) and Praz-Rodet bog in the Jura Mountains of Switzerland (46°34′ N 6°10′ E). An authorization (No 1449) was delivered by the “Service forêts de la faune et de la nature du canton de Vaud” (state office for nature conservation) for sampling in Praz-Rodet for 2011 and 2012 (January 2011 to end of summer 2012). The Duffey Lake *Sphagnum* sample was not collected within the Provincial Park and therefore, no permits were required. Cells were extracted from *Sphagnum* mosses through serial of filtrations, and then were washed 3 to 4 times with distilled water [23], [24]. We prepared seven independent extractions, four from *Archerella flavum* and three from *Amphitrema wrightianum*, each of these extractions contained between 50 to 70 cells. Both species were documented using light microscopy (Figure 1).

### DNA Extraction, PCR Amplification and Sequencing

DNA was extracted using a guanidine thiocyanate-based protocol [25]. Seven SSU rRNA sequences (four from *Archerella flavum* and three from *Amphitrema wrightianum*) were obtained by two amplifications. The first amplification was performed using universal eukaryotic primers, 1EKF (CTGGTTGATCCTGCCAG) and 1498R (CACCTA CGGAAACCTTGTTA) or 1520R (CYGCAGGTTCACCTA), in a total volume of 30 µl with amplification profile consisting of (5 minutes at 95°C followed by 40 cycles of 30 sec at 94°C, 30 sec at 58°C and 1 min 30 sec at 72°C with a final elongation of 10 min at 72°C). The positive products were cloned into pCR2.1 Topo TA cloning vector (Invitrogen) and transformed into *E. coli* TOP10’ One Shot cells (Invitrogen) according to the manufacturer’s instructions. Clone inserts were amplified with vector T7 and SP6 primers. The expected size clones from PCR amplifications were purified with the NucleoFasts 96 PCR Clean Up kit from Macherey-Nagel (Düren, Germany) and sequenced with an ABI PRISM 3700 DNA Analyzer (PE Biosystems, Genève, Switzerland) using a BigDyeTM Terminator Cycle Sequencing Ready Reaction Kit (PE Biosystems). We also designed the following primers for internal sequencing: Archer1F (GTAAATTACCCAATCCYAAMTCG), Archer1R (AAACATTTTGCTTTCGC), and Archer2R (TTTGTCCTGCCCTGCT). The positive products were cloned; and two to five clones from each extraction of *Archerella flavum* and *Amphitrema wrightianum* were sequenced. Sequences are deposited in GenBank with the Accession Numbers: *Amphitrema wrightianum* PR-1 (KC245091); *Amphitrema wrightianum* PR-2 (KC245092); *Amphitrema wrightianum* PR-2 (KC245093); *Archerella flavum* BC-1 (KC245094); *Archerella flavum* BC-2 (KC245095); *Archerella flavum* BC-3 (KC245096) and *Archerella flavum* BC-4 (KC245097).

### Alignment and Phylogenetic Analysis

All SSU rRNA gene sequences were submitted to BLAST [26] in order to check their similarity with other available data in Genbank. Related sequences together with our sequences were added to a recent dataset [27] and aligned using the BioEdit software [28]. Introns, insertions and variable regions in the SSU rRNA alignment that could not be aligned unambiguously were removed from the analyses. Phylogenetic trees were reconstructed using both Maximum Likelihood and Bayesian approaches based on 800 bp alignment using some sequences of Rhizaria as outgroup.

The maximum likelihood tree was built using RAxML version 7.2.8 algorithm [29] as proposed on the Black Box portal (http://phylobench.vital-it.ch/raxml-bb/) using the GTR+Г+I model. Model parameters were estimated in RAxML over the duration of the tree search. The obtained tree was compared to the one that built by Bayesian analysis using the software MrBayes v. 3.1.2 [30]. We performed two simultaneous MCMC chains, and 500,000 generations. The generations were added until standard deviation of split frequencies fell below 0.01 according to the manual of MrBayes 3.1. For every 1,000^th^ generation, the tree with the best likelihood score was saved, resulting in 10,000 trees. The burn-in value was set to 25%. Trees were viewed using FigTree (a program distributed as part of the BEAST package). In addition, we performed approximately unbiased (AU) tests [31] to evaluate the likelihood of different alternative topologies to the obtained tree (see Results section).

## Results

**Figure 2:**
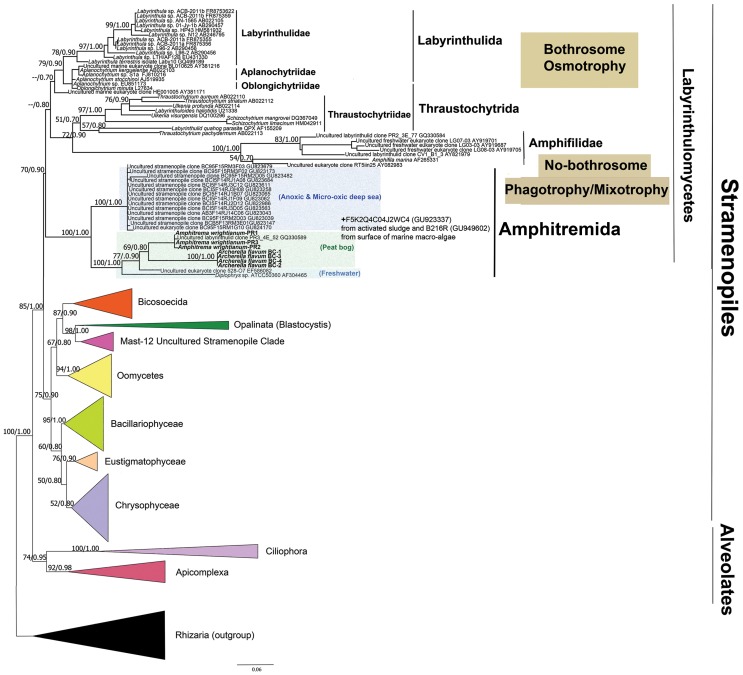
Molecular phylogenetic tree inferred from both maximum likelihood and Bayesian analysis based on small subunit (SSU) rRNA gene sequences and illustrating that the genera *Archerella* and *Amphitrema* (Amphitremida) belong to Labyrinthulomycetes (Stramenopiles). Numbers at nodes indicate the bootstrap values/posterior probabilities. Only values above 50/0.50 are shown. The tree was rooted with the group of Rhizaria. The scale bar indicates 0.06% sequence divergence.

We obtained seven SSU rRNA gene sequences, four from *Archerella flavum* and three from *Amphitrema wrightianum* with SSU rRNA gene length of 1287 bp and 1351 bp, respectively. The most similar SSU rRNA gene sequences to ours as revealed by BLAST were members of the labyrinthulida and thraustochytrida (Labyrinthulomycetes; Stramenopiles), plus the amoeboid *Diplophrys* and some environmental sequences. Therefore, we built an alignment that included some of the available SSU rRNA gene sequences of Labyrinthulomycetes/Labyrinthulea and other related taxa. Our constructed phylogenetic trees inferred from both RAxML and Bayesian analyses had the same topology (Figure 2). Labyrinthulomycetes receive a moderate support values (BS = 70%, PP = 0.90) and appeared divided into three major groups: (1) labyrinthulida+thraustochytrida, (2) a group comprising thraustochytrida*+*Amphifilidae (*Amphifila marina* and several freshwater environmental sequences) and (3) the group formed by *Archerella flavum* and *Amphitrema wrightianum* together with other related taxa: *Diplophrys* sp. ATCC50360(AF304465), and several environmental sequences, including PR3_4E_52 (GQ330589) from a peat bog, 528-O7 (EF586082) from freshwater, plus fourteen sequences from anoxic/micro-oxic deep-sea environment. As the position of *Paramphitrema* remains dubious [8], and because many possibly divergent organisms will be included in that clade, we name this third group Amphitremida, keeping Amphitremidae for the group comprising both *Amphitrema* and *Archerella*. As members of this group have traditionally been treated under the ICZN ( = Zoological nomenclatural code), this code will continue to being applied to it regardless of current or future phylogenetic placement [32]. As a result, we consider that family Diplophryidae, which has been described by Anderson and Cavalier Smith [33] cannot be valid, since it includes the environmental clone PR3_4E_52 (GQ330589), a sequence that belonged actually to *Amphitrema*. Amphifilidae and Amphitremida both received maximal support values (BS = 100%, PP = 1.00) (Figure 2). *Amphitrema wrightianum* and *Archerella flavum* plus clone PR3_4E_52 (i.e. Amphitremidae) formed together a moderately supported clade (BS = 77%, PP = 0.90). All the four obtained *A. flavum* sequences were exactly identical, while the obtained sequences of *A. wrightianum* PR-2, showed two nucleotides substitution at position 595 bp and 854 bp in comparison of both *A. wrightianum* PR-1 and PR-3 sequences. Our results have been confirmed by cloning the SSU rRNA fragments from each taxa.

The environmental peat bog clone PR3_4E_52 was also very closely related to these sequences and showed 99% similarity to *A. wrightianum* sequences. The freshwater environmental sequence 528-O7 (EF586082) had a basal position with respect to *Archerella flavum* and *Amphitrema wrightianum*. Fourteen SSU rRNA sequences from marine anoxic and micro-oxic water column branched as a sister clade to the peat bog+freshwater clade.

Following up on these results, we performed approximately unbiased (AU) test [31] to test the following alternative hypotheses: a) the monophyly of ((Amphitremida+Amphifilidae)+(thraustochytrida+labyrinthulida)), b) the monophyly of ((thraustochytrida+labyrinthulida+Amphifilidae)+(Amphitremida)). The tests did not reject any of these hypotheses (with *p-*values = 0.49 and 0.48 respectively).

## Discussion

### 1. Phylogenetic Position of Genera *Amphitrema* and *Archerella*, and Evolution of the Labyrinthulomycetes

This study demonstrates that the genera *Archerella* and *Amphitrema* belong to the Labyrinthulomycetes/Labyrinthulea (Stramenopiles) (Figure 2), rather than to other filose testate amoeba such as Euglyphida, *Pseudodifflugia* (Cercozoa), or the Foraminifera. Filose pseudopodia therefore appeared several times in eukaryotic evolution, not only within Rhizaria but also in some Opisthokonts (Nuclearia; see [34]) and Heterokonts (*Leukarachnion*; see [35]) and now, Amphitremida.

The labyrinthulida and thraustochytrida are characterized by the presence of a bothrosome or a ‘sagenetosome’ (sometimes also called ‘sagenogen’), an organelle that produces cytoplasmic networks (extensions of the plasma membrane) to absorb the nutrients from the surrounding environments, similar in that to fungi [36], [37]. Other genera such as *Labyrinthula* and *Aplanochytrium* also use these cytoplasmic networks for gliding [38]. The Labyrinthulomycetes are mainly osmotrophic protists. They are extremely common in marine environments, are often associated with decaying plants such as mangrove leaves [39], and less frequently parasitic [40]. The labyrinthulids and thraustochytrids exhibit a typical dimorphic life cycle with a vegetative absorptive stage and a flagellated zoosporic stage. Although genera *Archerella*, *Amphitrema*, *Diplophrys* and *Amphifila* move also by filose ectoplasmic extensions they do not possess a true bothrosome, and biflagellated stage have not yet been observed [14] (Table 1).

Our phylogenetic analysis suggests that the typical organisation with two symmetrical pseudopodial tufts found in *Amphitrema, Archerella, Diplophrys* and *Amphifila marina* might be ancestral to both Amphitremida and Amphifilidae. The AU test does not reject the existence of one clade grouping both Amphitremida and Amphifilidae and a second clade uniting the thraustochytrida together with labyrinthulida, an evolutionary pathway that appears the most parsimonious because it implies a single appearance of the bothrosome, a unique feature of thraustochytrida and labyrinthulida (Figure 2), and a simultaneous loss of pseudopodia and phagotrophy [38]. Under this evolutionary hypothesis, the bilateral symmetry of the cells would be a synapomorphy of Amphitremida+Amphifilidae, a character shared by all known members. Because osmotrophic state is not likely to be reversed back into a phagotrophic state (this would require regaining structures necessary for phagocytosis), we can hypothesize that the ancestral Labyrithulomycetes were phagotrophic and amoeboid organisms, possibly with a bilateral symmetry. In support to this interpretation, the basal-branching *Schizochytrium mangrovei* and *Thraustochytrium striatum* can shift from osmotrophic vegetative stage to phagotrophic amoeboid stage ingest through pseudopodia, if kept in culture together with bacteria, illustrating the dual nature of these organisms [41]. *Diplophrys* sp and *Amphifila marina* are both phagotrophic, and recent studies based on stable isotope ratios suggest that *Archerella flavum* is bacterivorous (Vincent Jassey, unpublished data), in contrast to earlier suppositions [20].

Another possible candidate for the assignment to both Amphitremida and Amphifilidae is *Sorodiplophrys stercorea*, an organism isolated from cow and horse dung with bilateral symmetry and filose pseudopodia, and devoid of bothrosome; interestingly, it relies entirely on osmotrophy [42].

### 2. Environmental Diversity of the Amphitremida

Our tree analysis including environmental clones revealed an unexpected diversity of organisms branching within Amphitremida that derived from a very wide range of environments (Figure 2). These include a freshwater biofilm clone from New Zealand, 528-O7 [43] that showed more than 98% similarity to our *Archerella flavum* sequences, and also, surprisingly, fourteen sequences obtained from anoxic and micro-oxic water column from the Cariaco Basin in the Caribbean Sea [44]. Their pervasive presence suggests that they are either genuine members of planktonic communities, or that they are associated to sinking debris in the water column. In addition, one pyro-tag from activated sludge F5K2Q4C04J2WC4 (GU923337) and one environmental sequence B216R from the surface of marine macro-algae (GU949602), both unpublished data from GenBank have been found to have high similarity with *Archerella* and *Amphitrema* respectively, but were not included in our analysis due to their short length. Nevertheless, these sequences further illustrate the diversity of habitats colonized by the hitherto unrecognised clade of Amphitremida.

The environmental sequence PR3_4E_52 (GQ330589) obtained by Lara et al., [27] from a eukaryotic diversity survey of the central pool in a pristine peat bog in the Swiss Jura Mountains branched together with our *Amphitrema wrightianum* sequences, from which it differs by three nucleotides at most. The different extractions of *A. wrightianum* also showed small differences in their sequences (up to two nucleotides). This diversity suggests the presence of several genotypes within the morpho-species *A. wrightianum* and/or the existence of several closely related taxa (cryptic species). Indeed, confusion with the very similar-looking peat bog species *A. stenostoma* [45] cannot be excluded. The two other described *Amphitrema* species (*A. lemanense* [46] and *A. congolense* [47]) do not possess endosymbiotic algae, and their phylogenetic position within the genus still needs to be determined. In contrast, our analyses did not reveal any intra-species genetic variability within *Archerella flavum*.

The Amphitremida represents a novel major clade within the Labyrinthulomycetes. This clade has colonised environments as divergent as peat bogs, freshwater and the oceans, ranging from nitrogen-depleted environments to eutrophic (sludge); their metabolisms vary from aerobic to anaerobic/micro-aerophilic, they can be phagotrophic or mixotrophic and have a planktonic or a benthic lifestyle. This illustrates the immense versatility of this group that certainly encompasses an even larger environmental genetic diversity than currently known. The true magnitude of this diversity will most probably be revealed by future massive environmental sequencing studies.
